# Functional, morphological and molecular characteristics in a novel rat model of spinal sacral nerve injury-surgical approach, pathological process and clinical relevance

**DOI:** 10.1038/s41598-022-13254-6

**Published:** 2022-06-15

**Authors:** Junyang Li, Shiqiang Li, Yu Wang, Aijia Shang

**Affiliations:** 1 The School of Medicine, Nankai University, Tianjin, 300071 China; 2 Department of Neurosurgery, General Hospital of Chinese People Liberty Army, No. 28 Fuxing Road, Beijing, 100853 China; 3 The 80Th Group Army Hospital of Chinese People Liberty Army, Shandong, 261021 China; 4 Institute of Orthopedics, 4th, Chinese People Liberty Army General Hospital, Beijing, China; 5 Co-Innovation Center of Neuroregeneration, Nantong University, Nantong, 226007 People’s Republic of China

**Keywords:** Bladder, Spinal cord diseases, Trauma, Experimental models of disease, Peripheral nervous system

## Abstract

Spinal sacral nerve injury represents one of the most serious conditions associated with many diseases such as sacral fracture, tethered cord syndrome and sacral canal tumor. Spinal sacral nerve injury could cause bladder denervation and detrusor underactivity. There is limited clinical experience resolving spinal sacral nerve injury associated detrusor underactivity patients, and thus the treatment options are also scarce. In this study, we established a spinal sacral nerve injury animal model for deeper understanding and further researching of this disease. Forty 8 w (week) old Sprague Dawley rats were included and equally divided into sham (n = 20) and crush group (n = 20). Bilateral spinal sacral nerves of rats were crushed in crush group, and sham group received same procedure without nerve crush. Comprehensive evaluations at three time points (1 w, 4 w and 6 w) were performed to comprehend the nature process of this disease. According to urodynamic test, ultrasonography and retrograde urography, we could demonstrate severe bladder dysfunction after spinal sacral nerve injury along the observation period compared with sham group. These functional changes were further reflected by histological examination (hematoxylin-eosin and Masson’s trichrome staining) of microstructure of nerves and bladders. Immunostaining of nerve/bladder revealed schwann cell death, axon degeneration and collagen remodeling of bladder. Polymerase Chain Reaction results revealed vigorous nerve inflammation and bladder fibrosis 1 week after injury and inflammation/fibrosis returned to normal at 4 w. The CatWalk gait analysis was performed and there was no obvious difference between two groups. In conclusion, we established a reliable and reproducible model for spinal sacral nerve injury, this model provided an approach to evaluate the treatment strategies and to understand the pathological process of spinal sacral nerve injuries. It allowed us to understand how nerve degeneration and bladder fibrosis changed following spinal sacral nerve injury and how recovery could be facilitated by therapeutic options for further research.

## Introduction

Peripheral nerve injury occurs worldwide and remains a problematic condition for doctors. Many previous studies on peripheral nerve injury have focused on the brachial plexus and sciatic nerves, and the functional characterization and underlying molecular mechanism have been clearly demonstrated^1–4^. However, injury to the spinal sacral nerve is sometimes overlooked compared with other peripheral nerves^5^.

The etiologies of spinal sacral nerve injury are not rare but common and expansive, such as spinal bifida^6^, tethered cord syndrome^7^, anterior sacral meningocele^8,9^, sacral canal cyst and sacral fractures^10–12^. Compared with other nerve injuries that usually cause motor dysfunction, the spinal sacral nerve is a mixed nerve comprising motor, sensory and autonomic nerves (parasympathetic nerves)^13^ that mainly innervate the bladder and bowel^5,14,15^. Therefore, damage to the autonomic nerve fibers in the spinal sacral nerve would lead to detrusor underactivity and cause neurogenic bladder^16,17^.

Some clinical studies have demonstrated that 35% of patients with transverse sacral fractures have spinal sacral nerve root transection injuries, and spinal sacral nerve injury-associated urinary tract symptoms can be long lasting^11,18^. For polytrauma patients with sacral fracture, many physicians are concerned only with trauma, hematoma and hemorrhage but ignore spinal sacral nerve examination. Patients who miss the early time window for depression of the spinal sacral nerve have severe low urinary tract symptoms in the late course^10,19^. Tethered cord syndrome can lead to increased tension along the spinal sacral nerve and intraneural pressure inside the spinal sacral nerve. Urodynamic tests in these patients suggest detrusor underactivity, elevated postvoid residual, or vesicoureteral reflux. More than half of patients with spina bifida or tethered cord syndrome exhibit serious urinary tract symptoms^6,20^. Early and timely release can improve the quality of life and prevent the progression of urinary system damage^7,21^. Additionally, sacral canal cysts, lumbosacral region tumors and iatrogenic factors (pelvic surgery) can all cause acute or chronic spinal sacral nerve injury^8,22–24^. Spinal sacral nerve injury, therefore, is not uncommon. The implementation of multidisciplinary cooperation is necessary, and the underlying pathophysiological mechanisms of the disease and potential treatment strategies require further exploration.

Detrusor underactivity, although not unusual in clinical practice, has not been thoroughly evaluated. Most prevalent animal model studies related to lower urinary tract dysfunctions (LUTDs) have focused on overactive bladder, but a few DU models have been reported^25^. In 2002, the International Continence Society (ICS) defined detrusor underactivity (DU) as a reduced strength and duration of contraction of the detrusor muscle. Left untreated, long-lasting neurogenic bladder can cause urinary tract infection, hydronephrosis or even renal failure^26^. Etiology is multifactorial, including myogenic dysfunction or neurogenic injuries^27^. Regarding neurogenic factors, most previous studies have focused on central nervous system injuries, such as lumbosacral spinal injuries^28^.

However, in order to enable an exact evaluation of new therapeutic options for SSNI (spinal sacral nerve injury), it is important to adopt a reliable and reproducible animal model that mimics the real clinical symptoms. However, to our knowledge, a clinically relevant animal model for SSNI-DU has not been published yet. Pathophysiological changes and available treatment strategies for DU remain unclear, although it is not rare in clinical circumstances and warrants more attention than before^29^. We therefore developed a reliable rat model that exhibits the essential pathological processes of peripheral nerve scarring.

**Figure 1:**
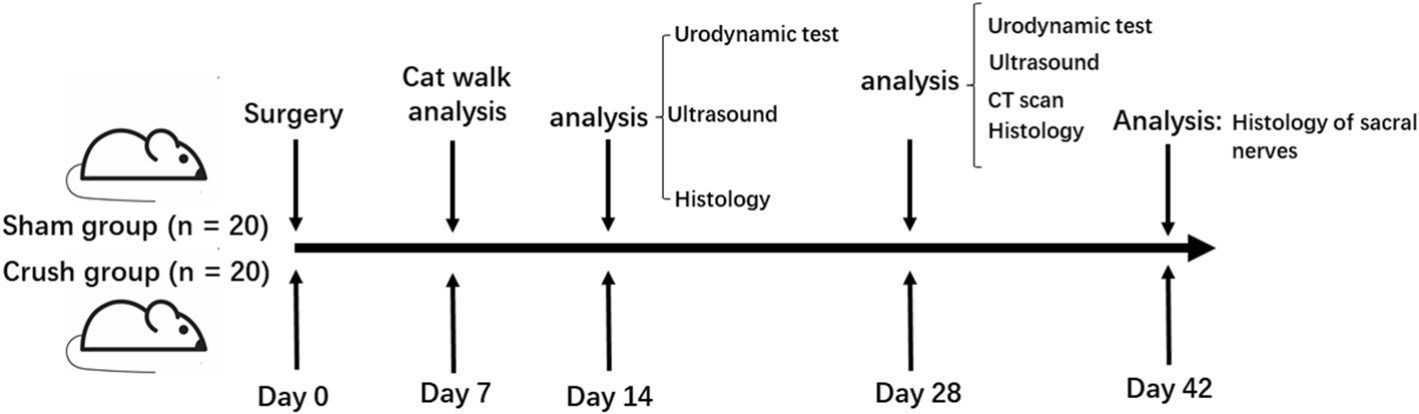
Study design. Experimental workflow for spinal sacral nerve injury in female rats.

**Figure 2:**
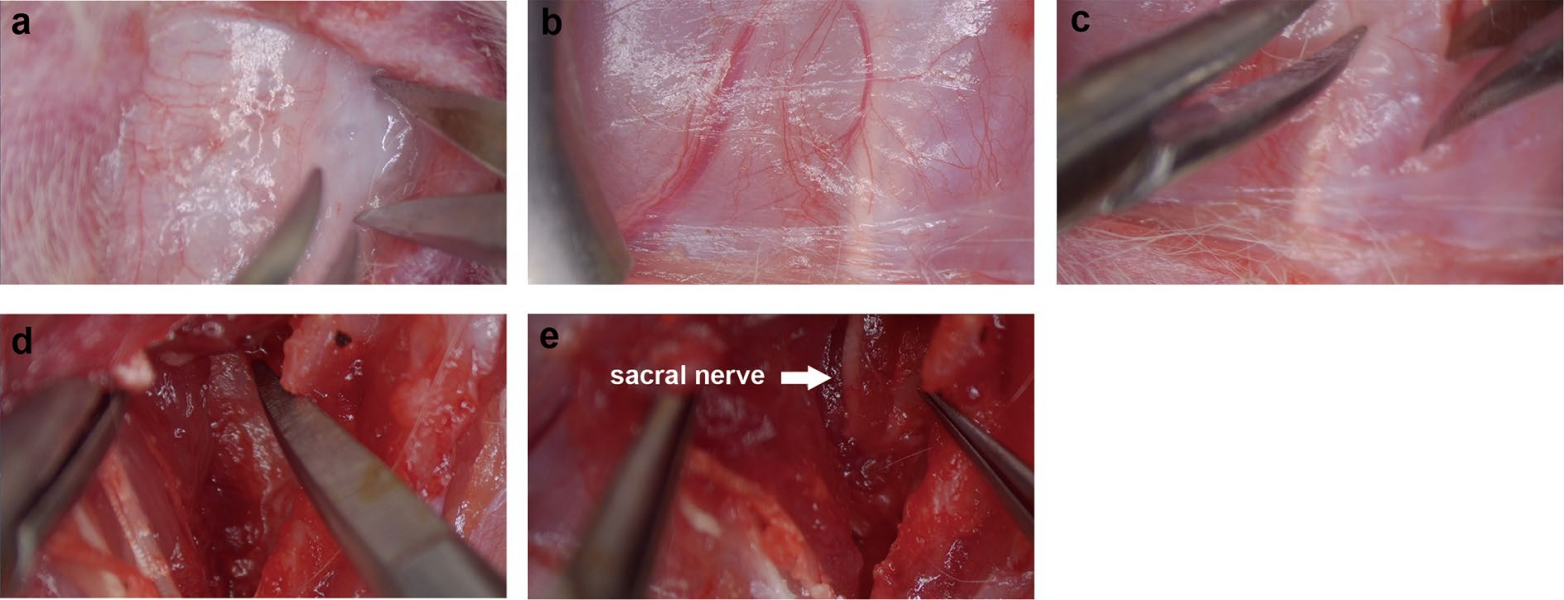
Surgical procedure of spinal sacral nerve injury. (**a**-**c**) Skin was dissected and spinal process was identified, paravertebral muscle was separated from vertebral. (**d**) Dorsal sacral foramen was identified. (**e**) Spinal sacral nerve 2 was exposed.

To our knowledge, for the first time, we created a new injury animal model with detrusor underactivity by crushing the bilateral spinal sacral nerves with micro forceps. The crush site of the spinal sacral nerve is at the root near the foramen where the spinal nerve exits the vertebra to ensure operations under similar clinical conditions, mimicking the clinical scenario of spinal sacral nerve injury, such as spinal bifida, sacrum fracture and certain iatrogenic factors. We also performed a full assessment to evaluate the characteristics of the SSNI model from functional, imaging, morphological and molecular level aspects (Figs. 1 and 2).

## Results

### Urodynamic testing

**Figure 3:**
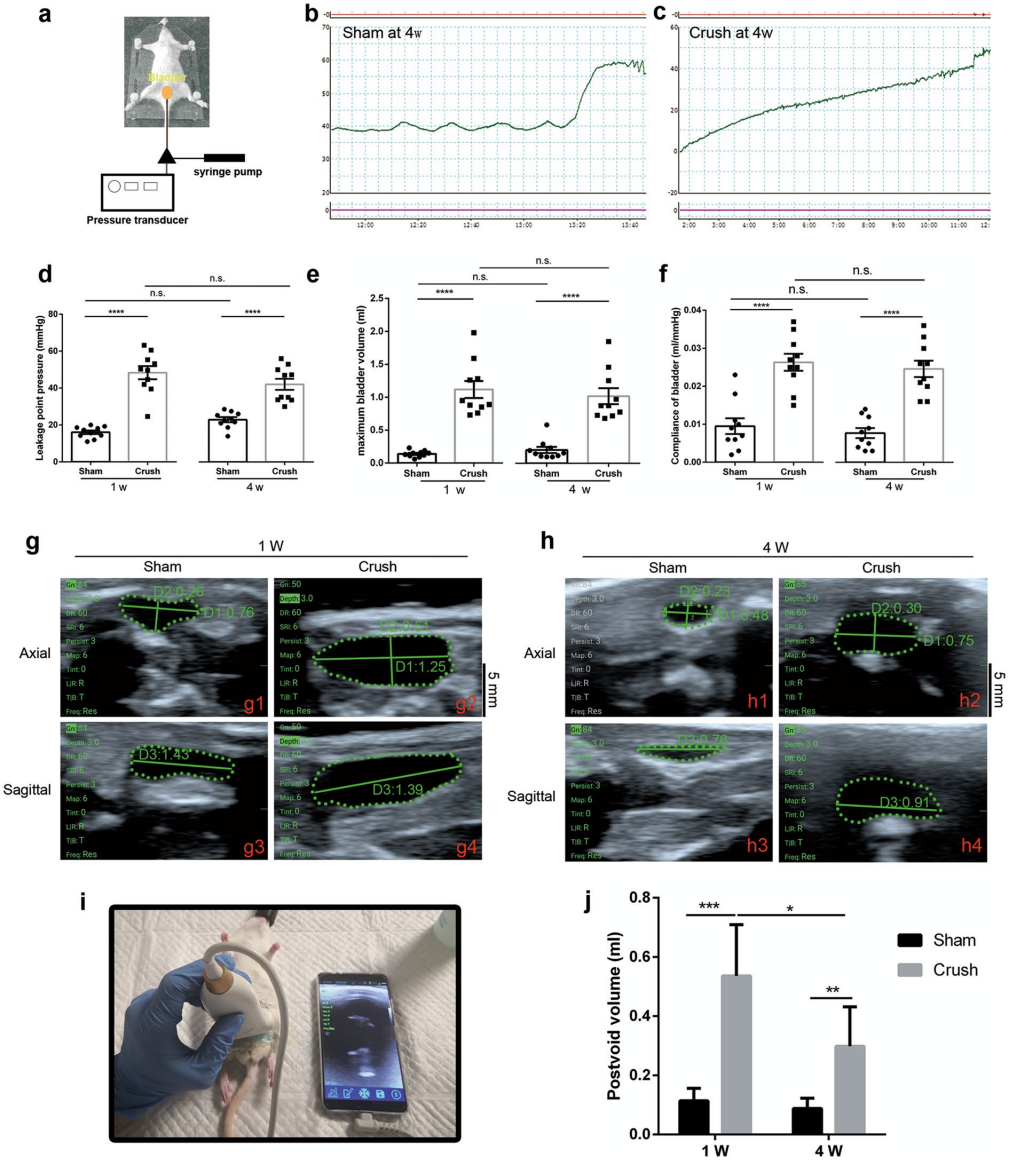
Results of urodynamic test and ultrasound examination at 1 week and 4 weeks. (**a**) Illustration image of urodynamic test procedure. (**b** and **c**) P–T curve of urodynamic test at 4 w. (**d**-**f**) Leakage point pressure, maximum bladder volume and compliance of bladder in two groups 1 week and 4 weeks after injury. N = 10/group. (**g** and **h**) Ultrasound axial and sagittal image of bladder in two groups at 1 w and 4 w. Ultrasound test was performed to measure bladder width and depth at axial position, length was measured at sagittal position. Scale bar: 0.5 cm. (**i**) Image of the ultrasound procedure of rats. A 2 – 10 MHz linear array transducer was used to measure bladder volumes of rats in two groups under isoflurane anesthesia. (**j**) Postvoid volume calculated from ultrasonography at 1 w and 4 w. Data of **d**-**g** was represented as mean ± SEM. Significance levels were set at ^*^P < 0.05, ^**^P < 0.01, ^***^P < 0.001, ^****^P < 0.0001.

To examine whether crush injury of the bilateral spinal sacral nerves could impact voiding function, we performed filling and voiding urodynamic experiments as previously described by Zheng et al*.*^30^ (Fig. 3a). The urodynamic testing parameters showed dramatic differences between the groups (Fig. 3b to f). The leakage point pressure (LPP) in the crush group was much higher than that in the sham group at 1 week and 4 weeks (Fig. 3d; *p <* 0.0001). Additionally, the maximum bladder volume (MBV) of the crush group was significantly higher than that of the sham operation group at two different points (Fig. 3e p < 0.0001), a finding that was consistent with the LPP results. However, bladder compliance was significantly higher in the crush group than in the sham group (Fig. 3f; *p <* 0.0001). These results suggested that bilateral spinal sacral nerves crush injury could cause denervation (parasympathetic nerve fibers) of the detrusor muscle, indicating an underactive detrusor and retention of urine. No rats died following sham and crush surgery.

### Ultrasonography

Transabdominal ultrasound was performed to evaluate the urine volume (Fig. 3i). The bladder appeared as dark, hypoechoic, oval structures, and the surrounding tissue appeared as a bright, hyperechoic area (Fig. 3g and h). The bladder volumes were calculated according to the formulation mentioned in the Methods section. The bladder volumes of rats in the crush group (0.5360 ± 0.1730 ml, 0.2980 ± 0.1337 ml) were significantly higher than those of rats in the sham group (0.1140 ± 0.0422 ml, 0.0880 ± 0.0349 ml) at 1 w and 4 w (Fig. 3j; *p = *0.0007 < 0.001 and *p = *0.0094 < 0.01).

### Retrograde urography

**Figure 4:**
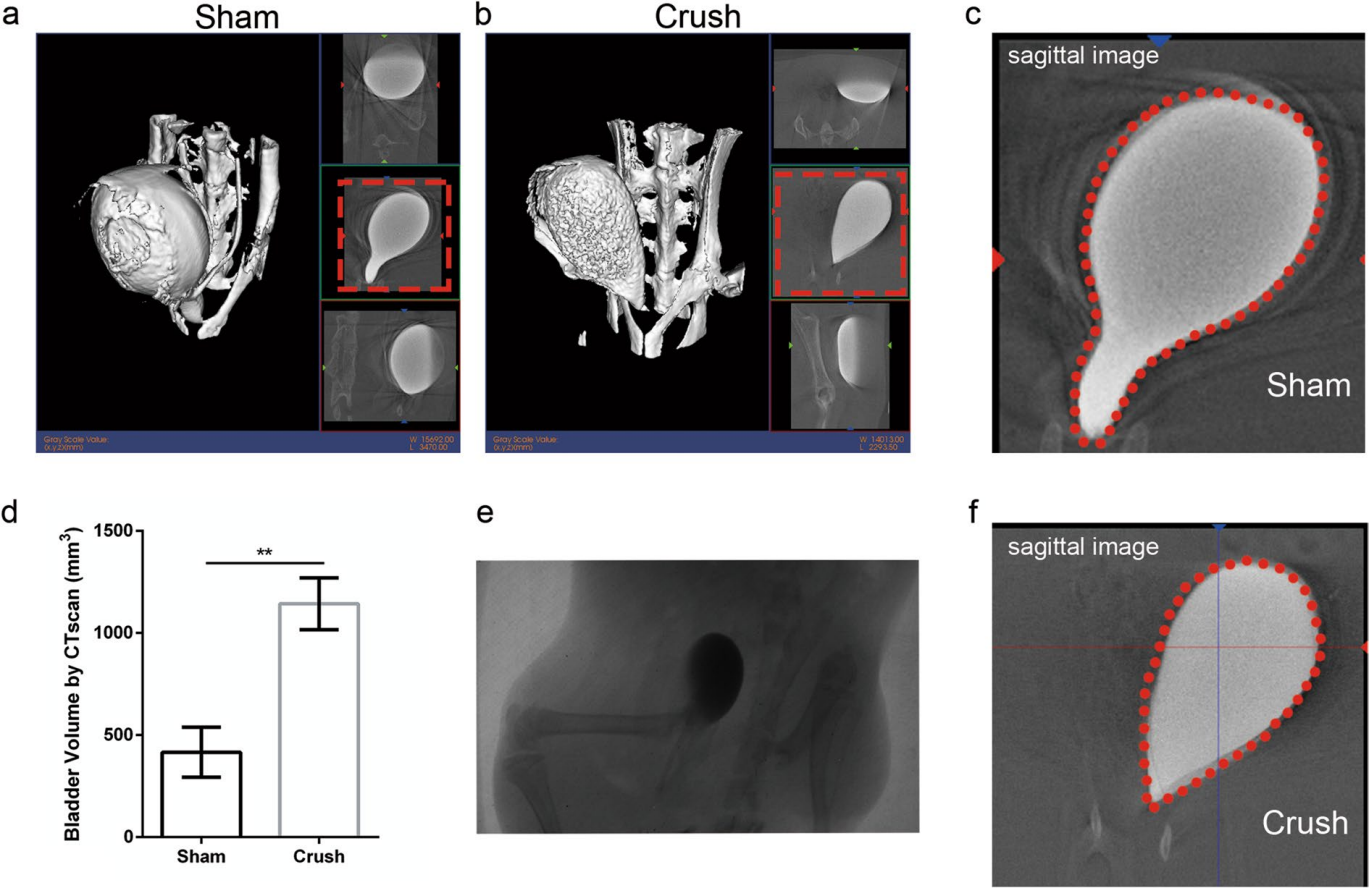
CT reconstruction of bladder and calculated volume. (**a** and **b**) 3-dimensional reconstruction of contrast media filled bladder of two groups, right inset images were axial, sagittal and coronal images from top to bottom, respectively. (**c**) CT fluoroscopy showed the real-time image of bladder. (**d** and **e**) Sagittal image of the contrast media filled bladder. (**f**) Bladder volume calculated by CT scan, N = 4/group. Data was represented as mean ± SEM. Significance levels were set at ^**^P < 0.01.

Sagittal, coronal and axial images of bladder-filled contrast medium are shown in Fig. 4a and b. Reconstructed bladder images are shown in the left panel. The outline of the maximal sagittal image sections is delineated using a red dotted line. The volume of the bladder was automatically calculated by the system. The bladder volume of rats in the crush group (1143 ± 253.9 ml) was significantly larger than that of rats in the sham group (415.7 ± 245.0 ml; Fig. 4f; *p = *0.0062 < 0.01). Morphological changes were observed in the crush group but not in the sham group (Fig. 4c, e and f).

### Gait analysis

**Figure 5:**
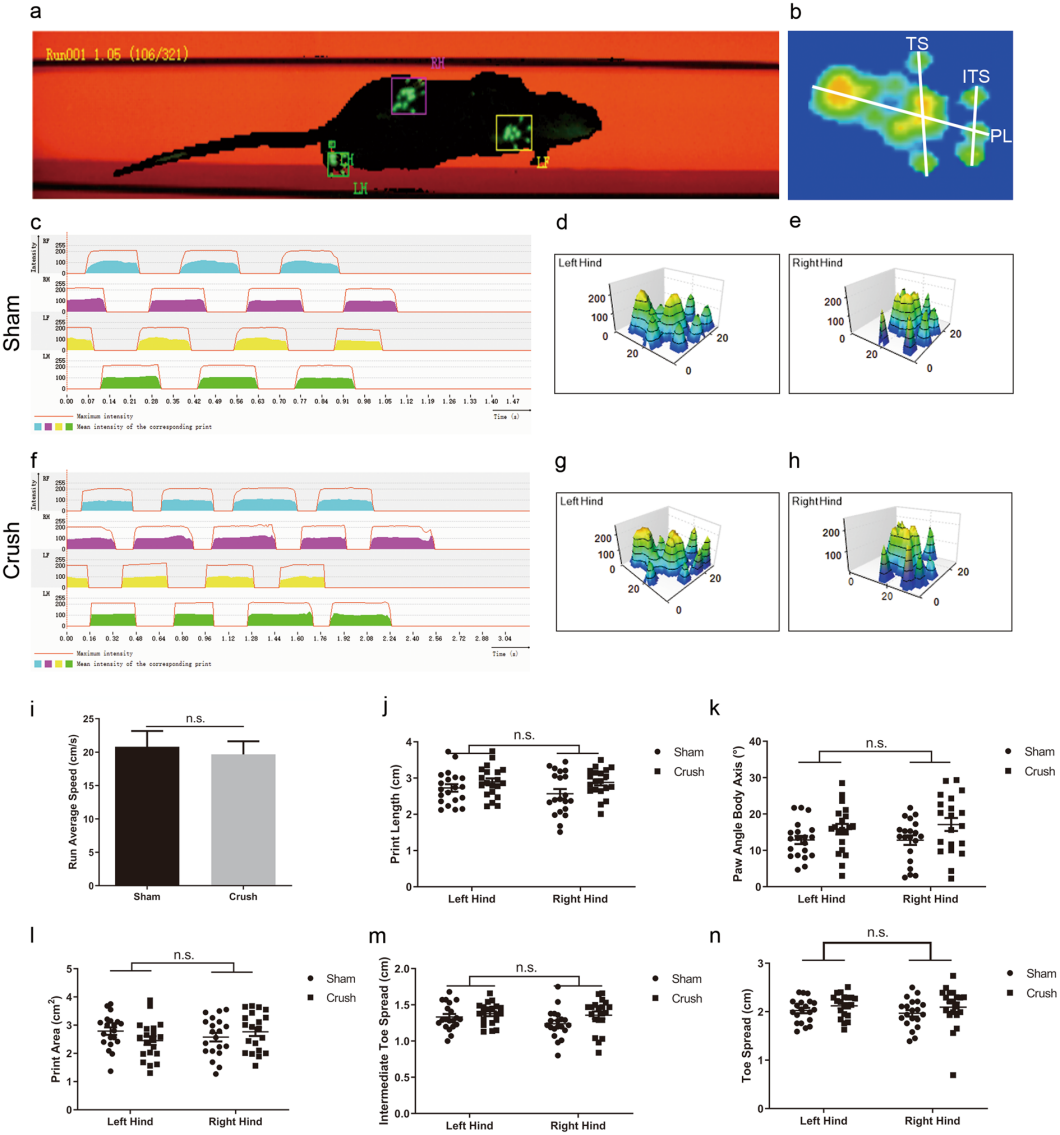
Gait analysis for judging the function of lower limbs in two groups. (**a**) Illustration of the walkway for gait analysis. (**b**) Examples of the footprint view and the measurements of toe spread (TS), intermediate toe spread (ITS) and print length (PL). (**c**-**h**) Examples of the 2D footprint intensities plot and 3D footprint intensities charts of rats in Sham and Crush group. The solid rad line represents the maximum intensity of the paw, the color-filled area under the rad line represents the mean intensity of the paw during the gait analysis. (**i**), Run Average Speed between two groups showed no statistical difference (N = 20/group). (**j**-**n**) Print Length (N = 20/group), Paw Angle Body Axis (N = 20/group), Print Area (N = 20/group), Intermediate Toe Spread (N = 20/group) and Toe Spread (N = 20/group) of two hinds between two groups all showed no significant difference. Homogeneity of variances was tested by using the Leneve's analysis before two-way-ANOVA. Data was represented as mean ± SEM. Significance levels were set at ^n.s.^*P* > 0.05.

To evaluate the locomotor function of rats in the two groups, we performed gait analysis (Fig. 5a to 5n). In many previous studies on animal models with lower urinary tract syndrome, the animals also had lower limb dysfunction, particularly in disease models such as contusive spinal injury models or conus medullaris transection models. However, in this study, no statistical difference of lower limbs movement was found in the run average speed (Fig. 5i; *p = *0.7060 > 0.05), print length (Fig. 5j; F = 2.215, *p = *0.4867 > 0.05), paw angle body axis (Fig. 5k; F = 2.999, *p = *0.6420 > 0.05), print area (Fig. 5l; F = 2.256, *p = *0.3827 > 0.05), intermediate toe spread (Fig. 5m; F = 2.036, *p = *0.6198 > 0.05) or toe spread (Fig. 5n; F = 2.001, *p = *0.0781 > 0.05) between the groups. The locomotor function of the lower limbs was not affected in the spinal sacral nerve crush model. These features were well correlated with the conditions from real clinical circumstances in which many patients with spinal sacral nerve injury only have symptoms of a lower urinary tract without obvious lower limb dysfunction.

### Histological evaluation of the bladders

**Figure 6:**
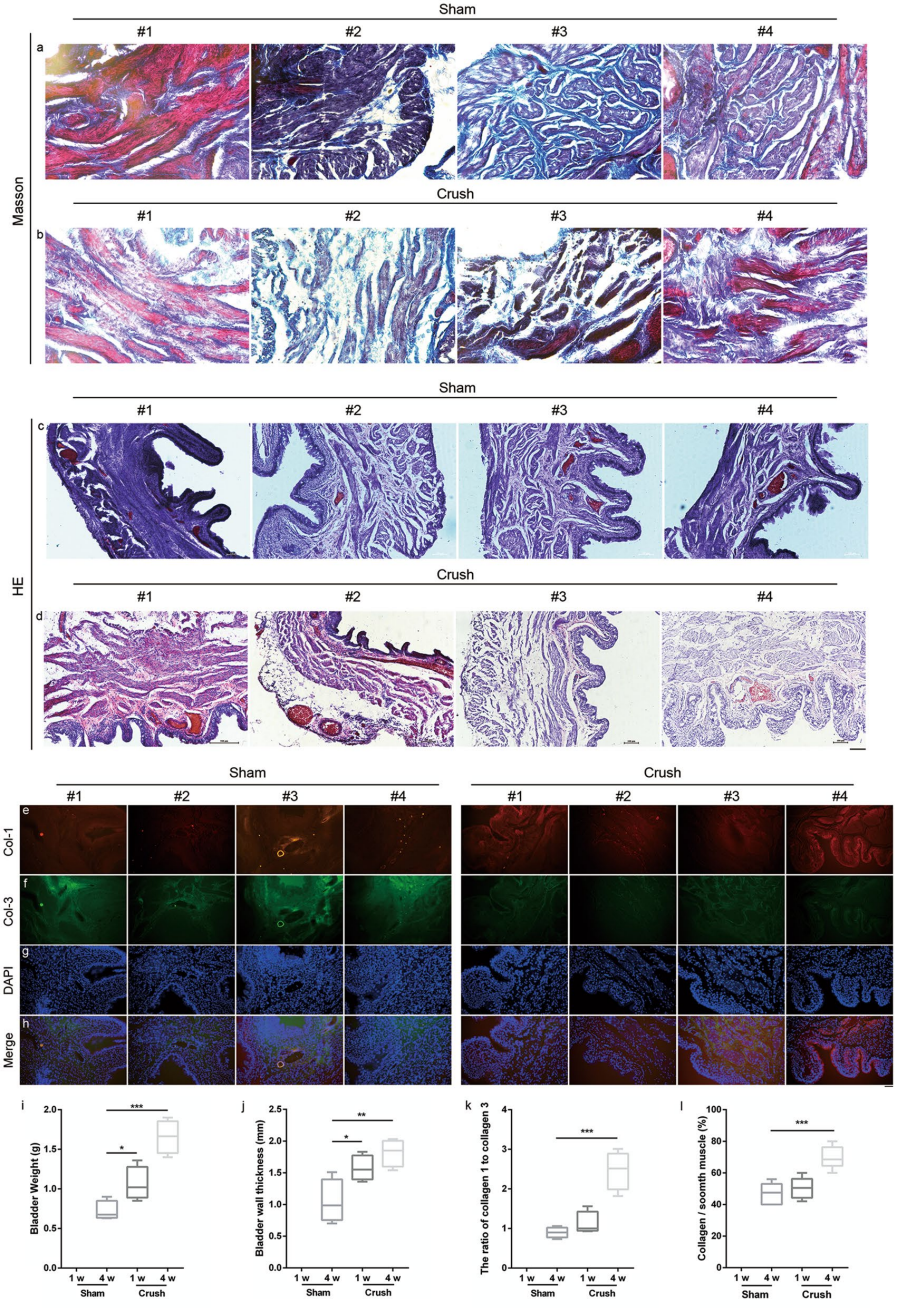
Histological evaluation and immunohistostaining of bladder at 1 w and 4 w. (a#1-a#4 and b#1-b#4) Masson’s trichrome staining of bladder wall (20 ×). (c#1-c#4 and d#1-d#4) H&E staining of bladder wall (10 ×) Scale bar: 100 μm. (e#1-e#4, f#1-f#4, g#1-g#4 and h#1-h#4 in sham; e#1-e#4, f#1-f#4, g#1-g#4 and h#1-h#4 in crush) Immunostaining of Col-1, Col-3, DAPI and Merge image of bladder (20x). Scale bar: 100 μm. (i-l) Bladder weight, bladder wall thickness, the ratio of collagen 1 to collagen 3 and collagen/smooth muscle at 1 w and 4 w. Data was represented as mean ± SEM. Significance levels were set at ^*^P < 0.05, ^**^P < 0.01, ^***^P < 0.001.

Histological analysis of Masson’s trichrome staining at 4 weeks after surgery showed an apparent increase in connective tissue and a decrease in muscle mass with the loss of normal tissue architecture (Fig. 6a#1 to a#4 and 6b#1 to b#4). H&E staining showed apparent bladder wall thickening and atrophy detrusor in the crush group compared with that in the sham group (Fig. 6c#1 to c#4 and 6d#1 to #4; *p <* 0.01, *p <* 0.01). Under a microscope at high magnification, vasodilation and inflammatory infiltration in the bladder wall were clearly observed in the crush group but not in the sham group (Fig. 6c#1 to c#4 and 6d#1 to d#4). Meanwhile, the immunofluorescence of collagen 1/3 demonstrated a collagen shifting after nerve injury (Fig. 6e#1 to #4, 6f.#1 to #4, 6 g#1 to #4 and 6 h#1 to #4), The weight of the bladder in the crush group was increased at 1 week and almost doubled at 4 weeks compared with that in the sham group (Fig. 6i; *p <* 0.05 and *p <* 0.001). The bladder wall thickness was significantly increased in Crush group compared with that in the sham group (Fig. 6j; *p <* 0.05 and *p <* 0.01). The ratio of collagen 1 to collagen 3 was significantly increased at 4 weeks (Fig. 6k, *p* < 0.001). Additionally, the ratio of connective tissue to muscle mass in the crush group was significantly larger than that in the sham group at 4 weeks not at 1 week (Fig. 6l; *p <* 0.001).

### Histological evaluation and immunofluorescent staining of the spinal sacral nerves

**Figure 7:**
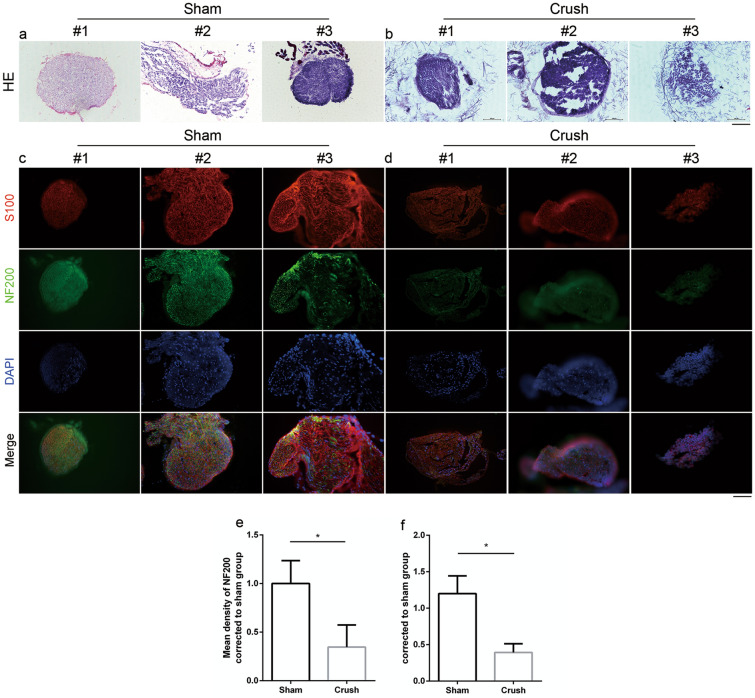
Histological evaluation and immunohistostaining of spinal sacral nerve at 1 w and 4 w. (a#1-a#3 and b#1-b#3) H&E of nerve in two groups. (c#1-c#3 and d#1-d#3) S100 and NF200 immunohistostaining of spinal sacral nerve in sham (20 ×) and crush group (20 ×). (**e** and **f**) Mean density of NF200 and mean density of S100 in two groups. Data was represented as mean ± SEM. Significance levels were set at ^*^P < 0.05.

To further evaluate the axonal degeneration and demyelination of the spinal sacral nerve, H&E staining and immunohistostaining was conducted to show Schwann cells and axons of the spinal sacral nerve. H&E showed the loss of normal structure of spinal sacral nerves compared to the nerves in sham group (Fig. 7a#1 to a#3 and b#1 to 7#3). Immunofluorescent staining showed that the density of NF-200 immunopositive nerve fibers in the sham group was much higher than that in the crush group. Additionally, the density of S100-positive SCs was significantly larger than that of the crush group (Fig. 7c#1 to c#3, 7d#1 to c#3, e and f; *p <* 0.05, *p <* 0.05). The images of immunofluorescence staining (NF200 and S100) of spinal sacral nerves at 6 week were shown in supplementary materials (Fig. 1S#1, #2 and #3). These findings suggest apparent degeneration and demyelination of spinal sacral nerve fibers in the crush group.

### Dynamic detection of local inflammation in nerves and fibrosis process in bladders after spinal sacral nerve injury

**Figure 8:**
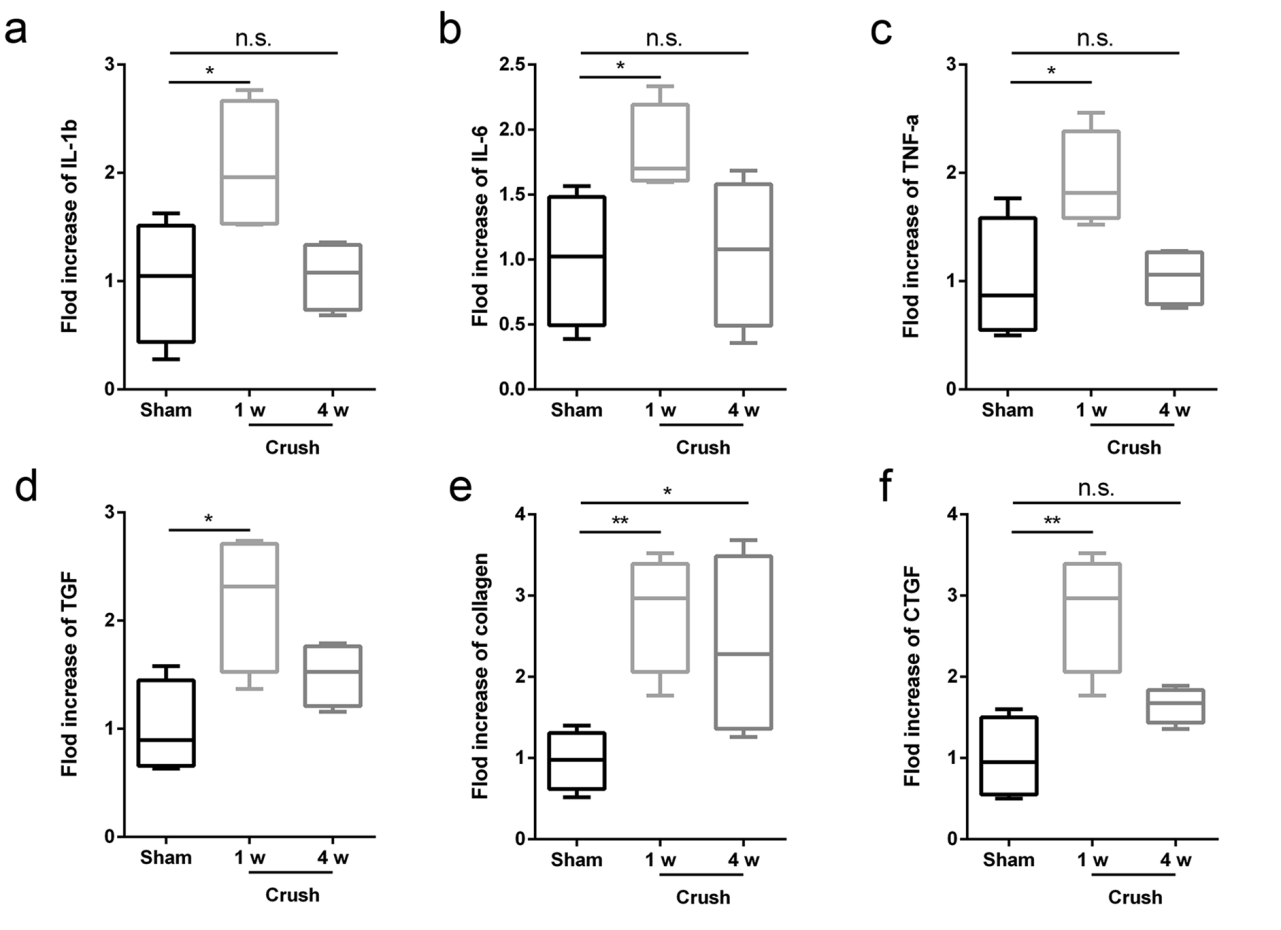
PCR analysis of pro-inflammation factors in nerve and pro-fibrosis factors in bladder. (**a**-**c**) Pro-inflammation factors (IL-1b, IL-6 and TNF-a) expression in sham group and crush group (1 w and 4 w). (**d**-**f**) Pro-fibrosis factors (TGF, collagen and CTGF) expression in sham group and crush group (1 w and 4 w). Data was represented as mean ± SEM. Significance levels were set at ^n.s.^*P* > 0.05, ^*^P < 0.05, ^**^P < 0.01.

In order to understand the inside mechanism in nerves and bladders after spinal sacral nerve injury, we did polymerase chain reaction (PCR) examination at 1and 4 week. PCR results showed that there was a significantly elevated expression of pro-inflammation factors (IL-1b, IL-6 and TNF-a) in local environment of spinal sacral nerve 1 week after injury. However, the expression of all pro-inflammation factors got back to normal level at 4 weeks after injury (Fig. 8a, p < 0.05, p > 0.05; 8b, *p <* 0.05, *p* > 0.05; and 8c, *p <* 0.05, *p* > 0.05). It was same with the PCR results in bladder, PCR results showed that the expression of pro-fibrosis factors (TGF, collagen and CTGF) was elevated at 1 week after injury and returned to normal at 4 week (Fig. 8d, p < 0.05, *p* > 0.05; 8e, *p <* 0.05, p > 0.05 and 8f., *p <* 0.05, *p* > 0.05).

## Discussion

Peripheral nerve injury has always been the focus of clinical studies and basic medicine^31,32^. However, regarding basic medicine research, most previous studies have focused on peripheral nerves such as the sciatic nerve^33^, brachial plexus^34^ and facial nerve^35^. Animal models of peripheral nerve injury and evaluation methods are mature^36,37^. However, to our knowledge, few studies have investigated spinal sacral nerve injury although injury to the spinal sacral nerve is not rare in “real-world” clinical realities^11,38^. The reasons for spinal sacral nerve injury are multifactorial and complex and include trauma, sacrum fracture, tethered syndrome, and sacral canal cysts^39^. In these cases, TCS (tethered cord syndrome) can cause traction injury, the mass effect of meningocele and cysts can cause compression injury to spinal sacral nerve, in some cases of sacral fracture, the end of fracture can cause acute crush or even total resection injury to spinal sacral nerve from S-1 to S-4 and the late formation of callus around sacral foramina can cause chronic compression injury to spinal sacral nerves^5^. The spinal sacral nerve, which comprises the parasympathetic component, innervates the detrusor and internal sphincter. Paralysis of the spinal sacral nerve can cause an underactive detrusor, directly leading to uroschesis. Uroschesis can severely influence the quality of life of patients, and secondary hydronephrosis and infection are nonnegligible and can be lethal. However, clinical research and animal studies on spinal sacral nerve injury-mediated underactive detrusor are lacking^40^; future studies are warranted. To better understand the pathophysiological process of spinal sacral nerve injury-mediated underactive detrusor, we used adult SD rats to establish a new animal disease model of spinal sacral nerve injury with overall evaluation, including functional, morphological, imaging and histological assessment.

Thirty years ago, Dennis published a retrospective analysis of sacral fracture. His article analyzed 236 clinical cases of sacral fracture and classified them into three subgroups: zone I (entirely lateral to the neuroforamina), zone II (involving the neuroforamina but not the spinal canal) and zone III (extending into the spinal canal). Dennis emphasized the importance of cystometrograms in patients with sacral fractures because the neurogenic bladder is an important complex caused by sacral fractures, particularly fractures in sacrum zone III, and is the complex most likely to be overlooked^38^. Miriam Y. Kim et al. pointed out that a special condition of sacral fracture is transverse sacral fracture. This uncommon injury type is frequently associated with severe neurologic deficits in the form of bowel/bladder disturbance^18^. However, all the clinical studies on spinal sacral nerve injury caused by sacrum fracture were serial case reports or descriptive studies; clinical and in-depth mechanistic studies on the pathophysiological course of spinal sacral nerve injury-mediated neurogenic bladder must be conducted. In addition to the damage to the spinal sacral nerve from direct violence injury, such as sacrum fracture, other factors can cause spinal sacral nerve injury and neurogenic bladder. We established a new animal disease model according to the new surgical procedure and evaluated the model from multiple perspectives.

Urodynamic testing is the gold standard method to diagnose neurogenic bladder disease^41^. Urodynamic tests in rats after bilateral spinal sacral nerve crush injury demonstrated a significant increase in leak point pressure and the maximum bladder volume at 1 and 4 weeks (Fig. 3d, e). Urodynamic tests also suggested an increase in bladder compliance in the crush group at 1 and 4 weeks (Fig. 3f). These results were similar to those from other DU models^2,30,42^. We speculated that the crush damaged the parasympathetic nerve fibers in the spinal sacral nerve. The consequent denervation caused an underactive detrusor, leading to weak voiding contraction. Therefore, bladder compliance was increased in the crush group^42^. However, this deduction was based on the observed value at 4 weeks; if the observation period was extended, we considered that the compliance of the bladder in the crush group was highly likely to decrease because of the development of fibrosis: a dynamic process may exist because of the diphasic action from denervation at an early stage and the subsequent fibrosis process^43^.

In addition to the urodynamic functional evaluation of the bladder, we also assessed the morphological characteristics of the bladder from imaging aspects using ultrasound and CT scans. Ultrasound (US) imaging technology and retrograde urography are currently widely used for bladder imaging. Ultrasound is available and convenient and widely used to measure postvoid residual (PVR) in clinical practice^44^. Many researchers have demonstrated the same applicability of ultrasound in animal studies as well^45,46^. Hans S. Keirstead et al. explored the feasibility of a noninvasive ultrasonographic method for bladder function in spinal contusive injury rats, and ultrasonography could accurately document bladder function^47^. Other studies also supported the feasibility of ultrasound to evaluate bladder function or residual urine volume in animal studies^48,49^. We used ultrasound to assess bladder function after spinal sacral nerve injury, and the results indicated that spinal sacral nerve injury rats had significantly larger bladder volumes than rats in the sham group throughout the observation period at 1 w and 4 w post-surgery (Fig. 3j).

Retrograde urography is also a useful tool to assess the morphological characteristics of the bladder. Many animal studies have demonstrated the effectiveness of retrograde urography^50,51^. We referred to the procedures from previous studies concerning retrograde urography and repeated them in this study. The data revealed that the bladder volume in the crush group was significantly larger than that in the sham group (Fig. 4), a finding that was consistent with the ultrasonography results. In addition to the quantitative results, such as the volume data, we also found some nonnegligible morphological changes in the denervation bladder in the crush group, likely indicating dysfunction of the parasympathetic nerve and long-lasting urinary retention^52^.

Urinary retention is a complex of lower urinary tract symptoms (LUTSs) that is caused by detrusor underactivity (DU). Patients can have symptoms, including intermittency, the feeling of incomplete bladder emptying and slow stream. The prevalence of DU ranges between 9 and 48% in men and 12% and 45% in women^53–55^. Previously, many researchers had performed meaningful trials to explore how to establish an underactive detrusor animal model. Xin Zheng et al. reported a type of detrusor underactivity model in rats by transecting the conus medullaris^30^. Ozsoy O et al. described the functional and morphological changes in the neurogenic bladder and locomotor dysfunction of spinal cord compression in an animal model^56^. Noritoshi Sekido et al. reported an animal model of underactive bladder using lumbar canal stenosis (LCS)^57^. Karel Dewulf et al. represented the functional and molecular characteristics of a detrusor underactivity rat model using bilateral pelvic nerve crush injury^58^. However, what is the difference between us and what’s new about us: in Zheng’s model, rats exhibited not only detrusor underactivity but also severe lower limb paralysis. Conversely, in the clinical condition, for example, patients diagnosed with tethered spinal syndrome could manifest severe neurogenic bladder symptoms but mild lower limb paralysis. Therefore, the data could not be used by researchers who want to explore pathophysiological processes and new treatment strategies for spinal sacral nerve injury-mediated neurogenic bladder. A similar situation was observed concerning the results from Ozsoy O and Noritoshi Sekido: the animal model with spinal injury or lumbar spinal canal stenosis manifested urine retention and severe intermittent claudication. In this study, we used gait analysis to evaluate lower limb function between the groups, and no significant difference was found between them, indicating that the spinal sacral nerve crush injury model rats only exhibited detrusor underactivity without or, at least, with no obvious lower-limb paralysis (Fig. 5).

In another detrusor underactivity animal model of bilateral pelvic nerve crush injury^58,59^, the surgical procedure was different from ours. We used the posterior approach, but they used the transperitoneal approach. Second, the basic starting point was different: we wanted to explore an animal model that mimicked the clinical circumstances in which patients had urine retention because of spinal sacral nerve injury. However, the bilateral pelvic nerve injury animal model focused more on the complication from pelvic surgery: iatrogenic detrusor underactivity. The common situation was radical hysterectomy^60–62^.

According to the histological results, we described a decreased detrusor thickness and increased weight of the bladder in the crush group, a finding that was similar to previous studies^58,63,64^. Histological results of the spinal sacral nerve showed a severe decrease in myelinated nerve fibers, which may due to the acute inflammation. Acute and severe inflammation reaction in local environment always leads to cell death of schwann cell and axon degeneration, which was confirmed by PCR results and similar to many studies’ results. Dynamic examination (at 1 week and 4 weeks) showed increased expression of pro-inflammation factors at 1 week and the expression returned to normal at 4 weeks. Masson’s trichrome staining also showed an increased ratio of collagen to detrusor muscle, indicating the fibrosis process, which was confirmed by PCR. TGF and CTGF are two major factors which play important role in the fibrosis of many different tissues such as heart^65^, lung^66^, kidney^67,68^ and bladder^69^. PCR results in this study were similar to previous study’s results from Metcalfe PD et al. ^63^ However, there were still some differences between our results with Ge Q et al., which may due to the difference of methods between model establishment^42^. Fibrosis was not only due to the urine retention but also denervation, so there were two basic pathphysiological influences on the fibrosis of bladder. If we could repair the injured spinal sacral nerve in time, the fibrosis process may be limited, and the outcome would be better.

## Conclusions

**Figure 9:**
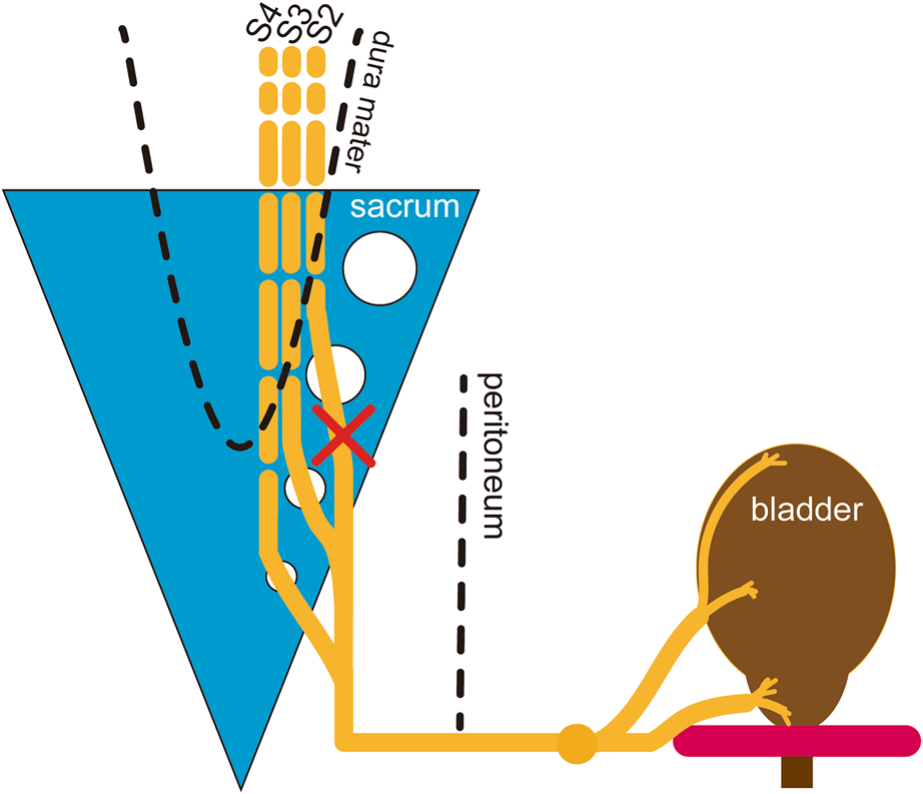
Schematic illustration of the establishment of SMI-DU rat model.

We established a SSNI animal model with detrusor underactivity using bilateral spinal sacral nerve crush injury (Fig. 9). We expounded on the clinical starting point of the establishment of this disease model and thoroughly evaluated the dynamic process pf functional, histomorphological and biomolecular characteristics of spinal sacral nerve injury-associated detrusor underactivity. In this pilot study, we provide a desirable and quantifiable animal model to further understand spinal sacral nerve injury and SSNI-associated detrusor underactivity.

## Methods

### Animals

The experimental procedures for the animals were performed according to the Guide for the Care and Use of Laboratory Animals from the Chinese Ministry of Public Health and the United States National Institutes of Health (Approval No. 2016- × 9–07). Forty healthy female Sprague–Dawley (SD) rats (8 weeks old, 200–225 g) purchased from the Chinese PLA General Hospital Animal Center were housed in groups (5 rats per cage) and bred under standard conditions of care with a 12-h day/night cycle, 40% humidity condition, free access to water and food and a temperature-controlled environment of 22 °C at the Experimental Animal Center of the Chinese PLA General Hospital. The rats were anesthetized with 3% sodium pentobarbital solution for animal surgery and isoflurane/urethane for functional and imaging analysis according to previously published articles. The rats were humanely sacrificed by cervical dislocation according to ARRIVE guidelines (Animals in Research: Reporting In Vivo Experiments).

### Study design

The rats were randomly divided into two groups (n = 20 rats/group): the sham operated group (Sham, n = 20) and the bilateral spinal sacral nerve crush injury group (Crush, n = 20). The primary evaluated outcomes were functional characteristics of the bladder using cystometrograms. Second, we evaluated the morphological characteristics of the bladder using multiple imaging tools, including computer tomography (CT) and ultrasound (US), and histological methods, including H&E and Masson’s trichrome staining. We also judged the injury level of the peripheral nerve (spinal sacral nerve) by histological methods, including the H&E and toluidine blue staining. We also used animal gait analysis to judge the dynamic posture and coordination during movement between the groups (Fig. 1).

### Spinal sacral nerve crush injury model

For surgery details, each rat was weighed and anesthetized via the injection of 3% sodium pentobarbital solution (2.5 mg/100 g of body weight). The back of the rats was then shaved, the spinal segment was localized on the surface, and an incision was made in the midline of the back of the rats. The dorsal sacral foramen was exposed after splitting the lumbar dorsal muscles. The bilateral spinal sacral nerves were exposed by removing part of the sacral bone, and the sacral bone was chipped away using a rongeur. The spinal roots of S1 and S2 could be identified under surgical microscopy (Fig. 2a–e). We induced crush injury (2 × 10 s) specifically on the bilateral root of the S-2 nerve using #WA2050 microneedle holders (Jin Zhong Inc., Shanghai, China). The overlying paravertebral muscles and skin were sutured in layers. Antibacterial spray was applied. After surgery, all the rats were placed on a preheated pad to re-establish thermoregulation. The sham group received the same operation without spinal sacral nerve crush injury. All the rats in the two groups were housed and fed a standard diet with ad libitum access to water and food. The bladders were emptied using the Crede procedure once daily until death. In the Crede procedure, gentle pressure is applied to the abdomen using two fingers. No rats died following sham and crush surgery.

### Urodynamic testing

The following urodynamic experiments were performed 1 week and 4 weeks after surgery. The rats in the two groups were anesthetized by urethane (1 g/kg), which is the most suitable anesthetic for studies related to micturition physiology^70^. In brief, a sterile polyethylene catheter (PE-50) was inserted into the bladder through the urethra. Intravesical pressure was measured using a PE-50 polyethylene catheter, which was connected via a three-way stopcock to an electrophysiology recorder (Millar, Texas, USA) with a pressure transducer, and pre-warmed normal saline was pumped into the bladder at a rate of 5.0 ml/h using an infusion pump (Harvard Apparatus, Cambridge, MA, USA) until the normal saline leaked from the external orifice of the urethra. Before each infusion, the bladder was compressed gently to empty the residual urine. The leakage point pressure (LPP) and maximum bladder volume (MBV) were collected as urodynamic measurement parameters, and bladder compliance was calculated according to the following formulation: bladder compliance = maximum bladder volume / (leakage point pressure – initial intravesical pressure).

### Micro-CT scan

Under anesthesia using urethane (1 g/kg) as previously described, iohexol solution was injected into the bladder using a PE-50 tube until the bladder was extended to the maximum volume under light anesthesia. Next, the rats were placed supine into a micro-CT machine for computer tomography urography at 4 w (General Electric Canada). A digital three-dimensional bladder (from external urethral orifice to the bladder dome) was reconstructed and bladder volume was calculated^50,71–73^.

### High-frequency microultrasonography

To measure the postvoid residual (PVR), we used a hand-held digital ultrasound imaging system (Lanmage, Shenzhen, China), a validated noninvasive diagnostic technique, to visualize the bladder of rats in the two groups at the end of the observation period after surgery. Ultrasound technology is a valuable translational method to measure PVR in animal research to quantify bladder dysfunction or functional recovery. Briefly, before scanning, the fur of the lower abdomen was shaved and removed using a depilatory paste. The abdomen was then cleaned with 70% ethanol and smeared with aquasonic gel (Parker Laboratories Inc., Fairfield, NJ), which was used to promote the transmission of sound through the skin to the bladder. Under isoflurane anesthesia (RWD Life Science Co., Shenzhen, China), the lower abdomen of rats was scanned using a linear probe (64 channels, 2–10 MHz, 38 mm). The morphological characteristics of the bladder were visualized, and the largest axial and sagittal sectional images were captured. The probe was first placed axially to capture the axial image and measure the width (D1) and depth (D2) of the bladder. Next, the probe was rotated 90 degrees to capture the sagittal image and measure the length (D3) of the bladder^47,49^. The volume was calculated according to the formula: volume (ml) = D1 × D2 × D3/2.

### Functional evaluation of lower limb muscle

To evaluate whether this new surgical procedure would impact the motor function of rats, we used the CatWalk XT 10.0 gait analysis system (Noldus, Wageningen, The Netherlands) 1 week after model surgery. The CatWalk analysis protocol has been described previously by published articlesyyyy^74,75^. Briefly, 40 rats (n = 20 per group) were included for gait analysis. The rats were placed on the right side of a runway covered by a glass surface with a black plastic background. A high-speed video camera was placed under the runway, and the run process of the rats crossing the glass runway was captured completely. The parameters, including the run average speed (RAS), print area (PA), toe spread (TS), intermediate toe spread (ITS), print length (PL) and paw angle body axis (PABA), were all recorded for further analysis. RAS is the speed of the animal’s body in the recorded run. PA is the surface area of the complete print. TS is the distance between the center of the first and fifth toes of a hind paw. ITS is the distance between the center of the second and fourth toes of a hind paw. MPL is the distance between the center of the third toe and heel of a paw. PABA is the smallest angle between the Manual Print Length line and line representing the orientation of the body axis.

### Histopathological evaluation

To further evaluate the bladder and nerve, the rats were sacrificed at 1 week, 4 week and 6 week (three time points) after surgery. The bladder and injury sites of the spinal sacral nerve were harvested, and the bladders were weighed (Mettler Toledo, Switzerland). As previously described, the nerves and bladders were fixed with 4% paraformaldehyde at 4 °C for 24 h. Thereafter, all the tissues were embedded in OCT Tissue Tek (Sakura, USA) and serially sectioned at 6-μm thickness. Subsequently, the bladder slices were subjected to hematoxylin–eosin (H&E) and Masson’s trichrome staining, and the nerve slices were stained with hematoxylin–eosin (H&E). After staining, light microscopic examination was conducted in tissue sections to evaluate the structure of the bladder and nerve (Olympus, Japan). The thickness of the bladder wall was measured, and the collagen tissue percentage was calculated as the ratio collagen/smooth muscle. The nerves were stained with hematoxylin and eosin (H&E) (Solarbio Life Sciences, Beijing, China). All the calculations and measurements were conducted using an image analysis system (ImageJ 1.50).

### Immunofluorescent staining

The tissue samples of spinal sacral nerves and bladders were immersed in OCT, frozen at -20 °C, and cut into 10-μm-thick sections. The nerve and bladder sections were then mounted on glass slides. Next, the slides were treated for 15 min with 0.1% Triton X-100 in PBS, and then blocked for 1 h in 10% normal goat serum (Cayman Chemical, Ann Arbor, MI, USA). After that, the nerve slides were incubated overnight with primary antibodies [rabbit anti-S100 polyclonal antibody (ab11428, 1:200; Abcam, Cambridge, UK) and mouse anti-neurofilament antibody (ab215903, 1:200; Abcam, Cambridge, UK)] at 4 °C, washed with PBS, and incubated with secondary antibodies [Alexa Fluor 488 goat anti-mouse (Abcam, Cambridge, UK) and Alexa Fluor 594 goat anti-rabbit (Abcam, Cambridge, UK)] at room temperature for 2 h. All the bladder slides were incubated overnight with primary antibodies [mouse anti-collagen 1 polyclonal antibody (ab88147, 1:200; Abcam, Cambridge, UK) and rabbit anti-collagen 3 antibody (ab6310, 1:200; Abcam, Cambridge, UK)] at 4 °C, washed with PBS, and incubated with secondary antibodies [Alexa Fluor 594 goat anti-mouse (Abcam, Cambridge, UK) and Alexa Fluor 488 goat anti-rabbit (Abcam, Cambridge, UK)] at room temperature for 2 h. All slides were finally covered with a cover glass. The slides were examined and imaged using a fluorescence microscope (Leica, Wetzlar, Hessen, Germany).

### Polymerase chain reaction (PCR)

Total RNA was isolated from spinal sacral nerves and bladders using an mirVana PARIS Kit (Life Technologies Corp.). According to the manufacturer’s instruction, isolated RNA was reverse-transcribed into cDNA by reverse transcription kit (Life Technologies Corp.). Then, the cDNA was amplified in a thermal cycler according to the following program: one minute for denaturation at 94°C, annealing at the primer specific (Table 1) temperature for one minute and following one minute for extension at 72°C. The products were amplified using 35 cycles for interleukin-1b (IL-1b), interleukin-6 (IL-6), tumor necrosis factor-a (TNF-a), transforming growth factor (TGF), Connective tissue growth factor (CTGF), collagen and glyceraldehyde-3-phosphate dehydrogenase (GAPDH), (Table 1.).

**Table 1:** List of primers used for reverse transcriptase-polymerase chain reaction.

Gene		Primer Sequence (5’-3’)	Temperature (°C)
*IL-1b*	Forward: Reverse:	CTCCATGAGCTTTGTACAAGG GGGGTTGACCATGTAGTCGT	58
*IL-6*	Forward: Reverse:	GAGTCCTTCAGAGAGATACAG CTGTGACTCCAGCTTATCTG	58
*TNF-a*	Forward: Reverse:	TCAGCCTCTTCTCATTCCTGC TTGGTGGTTTGCTACGACGTG	59
*TGF*	Forward: Reverse:	GGCTGAACCAAGGAGACGGAATAC GTGTGTCCAGGCTCCAAATGTAGG	58
*collagen*	Forward: Reverse:	TCACCAGACGCAGAAGTCATAGGAG GGAGACCACGAGGACCAGAAGG	58
*CTGF*	Forward: Reverse:	CATTAAGAAGGGCAAAAAGTGA CACACCCCACAGAACTTAGCC	56
*GAPDH*	Forward: Reverse:	GTCCATGCCATCACTGCCACTC CGCCTGCTTCACCACCTTCTTG	58

### Statistics

The data were presented as mean values ± standard deviation. Shapiro–Wilk test was adopted to ensure that all data were normally distributed. Student’s unpaired *t* test was used to test for significant differences between the groups. Two-way analysis of variance (ANOVA) plus a post hoc test was performed for several groups, homogeneity of variances was tested by using Levene’s test before two-way analysis of variance. P values < 0.05 were considered statistically significant. All the statistical analyses were performed using SPSS software 22.0 (IBM, USA).

### Ethical approval

All animal care and experimentation were performed according to the guidelines set by Chinese Ministry of Public Health and the United States National Institutes of Health. All animal studies were approved by the Guide for the Care and Use of Laboratory Animals from Chinese Ministry of Public Health (Approval No. 2016- × 9–07).

## Supplementary Information

Supplementary Information 1.

## Data Availability

All data, models, and code generated or used during the study appear in the submitted article.

## Abbreviations

SSNI: Spinal sacral nerve injury
LPP: Leakage point pressure
MBV: Maximum bladder volume
PVR: Postvoid residual
DU: Detrusor underactivity
LUTDs: Low urinary tract dysfunctions
ICS: International continence society
CT: Computer tomography
US: Ultrasound
PE: Polyethylene
RAS: Run average speed
PA: Print area
PL: Print length
TS: Toe spread
ITS: Intermediate toe spread
PABA: Paw angle body axis
H&E: Hematoxylin–eosin
TB: Toluidine blue
TCS: Tethered cord syndrome
