# Supplementary figures and images for: Functional, morphological and molecular characteristics in a novel rat model of spinal sacral nerve injury-surgical approach, pathological process and clinical relevance
Source: Sci Rep. 2022 Jun 15;12:10026. doi: 10.1038/s41598-022-13254-6 (PMC9200741; doi:10.1038/s41598-022-13254-6)

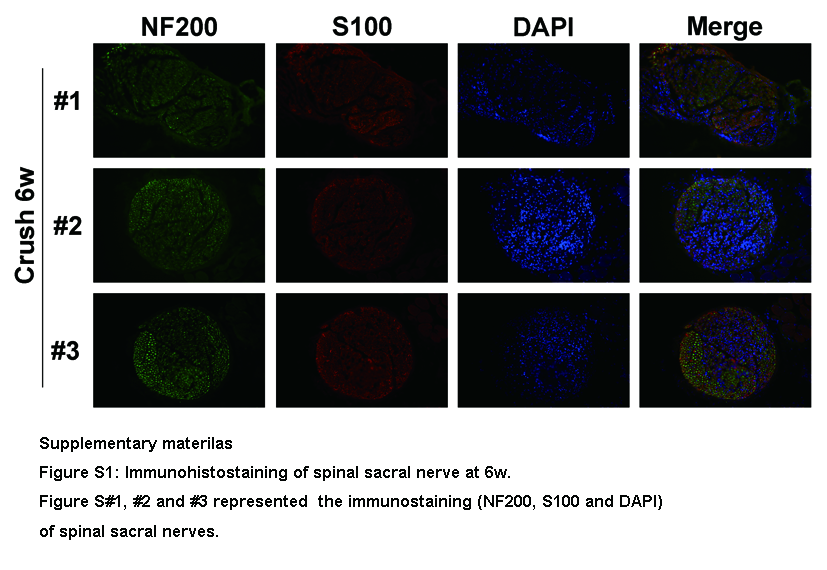

Supplement: Supplementary file 1 — Supplementary Information 1. [file 41598_2022_13254_MOESM1_ESM.tif]
